# Human Parainfluenza Virus Type 3 in Wild Nonhuman Primates, Zambia

**DOI:** 10.3201/eid1909.121404

**Published:** 2013-09

**Authors:** Michihito Sasaki, Akihiro Ishii, Yasuko Orba, Yuka Thomas, Bernard M. Hang’ombe, Ladslav Moonga, Aaron S. Mweene, Hirohito Ogawa, Ichiro Nakamura, Takashi Kimura, Hirofumi Sawa

**Affiliations:** Hokkaido University, Sapporo, Japan (M. Sasaki, A. Ishii, Y. Orba, Y. Thomas, H. Ogawa, I. Nakamura, T. Kimura, H. Sawa);; University of Zambia, Lusaka, Zambia (B.M. Hang’ombe, L. Moonga, A.S. Mweene)

**Keywords:** Human parainfluenza virus 3, viral RNA, viruses, serologic tests, nonhuman primates, Zambia

## Abstract

Human parainfluenza virus type 3 (HPIV3) genome was detected in 4 baboons in Zambia. Antibody for HPIV3 was detected in 13 baboons and 6 vervet monkeys in 2 distinct areas in Zambia. Our findings suggest that wild nonhuman primates are susceptible to HPIV3 infection.

Human parainfluenza viruses (HPIVs) (family *Paramyxoviridae*) are major causes of lower respiratory tract infections in infants and elderly persons. HPIVs are second to respiratory syncytial virus as the cause of hospitalizations for lower respiratory tract infections (*1*,*2*) and account for 6.8% of all hospitalizations for fever or acute respiratory illness in children <5 years of age (*3*). Among the 4 serotypes, HPIV3 (genus *Respirovirus*) causes particularly severe disease, including bronchiolitis and pneumonia (*1*–*3*). In addition to young children, HPIV3 poses a threat to the elderly and to immunocompromised adults. HPIV3 infection also causes severe illness leading to death (35%–75% death rate) in patients receiving hematopoietic stem cell transplants (*4*,*5*). Although the virus is distributed worldwide and maintained in human communities, its epidemiology in Africa is unclear.

Nonhuman primates, the closest living relatives of humans, are susceptible to paramyxoviruses that cause respiratory disease in humans. Recently, other researchers reported infections with human respiratory syncytial virus and human metapneumovirus in wild nonhuman primates in Africa (*6*,*7*). Therefore, as a first step in determining the pervasiveness of infection in African wild nonhuman primates, we screened these animals for paramyxovirus in Zambia. The HPIV3 genome was identified by seminested broad-spectrum reverse transcription PCR (RT-PCR). Thereafter, we investigated HPIV3 infection in wild nonhuman primates by using molecular and serologic methods.

## The Study

Baboons and vervet monkeys live side by side with humans in game management areas in Zambia, and this situation often leads to high levels of human–baboon/monkey conflicts. The Zambia Wildlife Authority is mandated by the Zambian government to control the large numbers of these animals. We collected tissues and serum samples from baboons and vervet monkeys killed for pest management purposes with the permission of the Zambia Wildlife Authority (certificate no. 2604). Samples were obtained from 50 yellow baboons (*Papio cynocephalus*) and 50 vervet monkeys (*Chlorocebus pygerythrus*) in the Mfuwe region (13°16’30.2”S, 31°40’00.4”E), Eastern Province, Zambia, in 2009 and from 50 chacma baboons (*P. ursinus*) and 39 vervet monkeys (*C. pygerythrus*) in the Livingstone region (17°50’8.72”S 25°43’59.19”E), Southern Province, Zambia, in 2010 and 2011. Sample information is summarized in the Table. The species were identified on the basis of morphologic characters and the mitochondrial cytochrome b (*cytb*) gene sequence. The complete *cytb* gene was amplified from spleen DNA of *Papio* spp. baboons with the primer set papio cytb1F (5′-GATACGAAAAACCATCGCTGT-3′) and papio cytb2R (5′-GCTCCATTTCTGGTTTACAAG-3′), as described (*8*), and from spleen DNA of *Chlorocebus* spp. monkeys with the primer set chlorocebus cytb1F (5′-TGATATGAAAAACCACCGTTGT-3′) and chlorocebus cytb2R (5′-GCTTTCTTTCTGAGTTGTCCTAGG-3′), designed in this study.

Total RNA was extracted from 189 spleen tissue samples by using TRIzol reagent (Life Technologies, Carlsbad, CA, USA) and screened for paramyxoviruses by seminested broad-spectrum RT-PCR of paramyxovirus polymerase (*L*) genes (*9*). Amplification was carried out with the primers PAR-F1, PAR-F2, and PAR-R. Seminested RT-PCR was positive in only 1 chacma baboon sample. The PCR product (584 bp) was subjected to direct sequence analysis, and the identified paramyxovirus was tentatively named ZMLS/2011. BLAST analysis (http://blast.ncbi.nlm.nih.gov/blast.cgi) indicated that ZMLS/2011 shared 98% nt identity with the HPIV3 *L* gene. To increase the sensitivity of HPIV3 genome detection, we screened all 189 RNA samples by RT-PCR, using the HPIV3 *L* gene–specific primer sets HPIV3 L1F (5′-ATGGGAGAATTCTTCCTCAAGCTC-3′) and HPIV3 L2R (5′-AATGCRGCAACTGATGGATCACC-3′). An HPIV3 genome was detected in 3 (6%) of 50 chacma baboon samples and in 1 (2%) of 50 yellow baboon samples but not from any of the 89 vervet monkey samples. Nucleotide sequences of all 4 amplicons (367 bp) were identical to ZMLS/2011.

In an attempt to isolate virus from RT-PCR–positive spleen, Vero cells cultured in minimum essential medium supplemented with trypsin were injected with tissue homogenates; however, after 3 passages, cytopathic effects were not observed. Viral RNA was also not detected in the culture supernatants from the cells.

**Figure 1:**
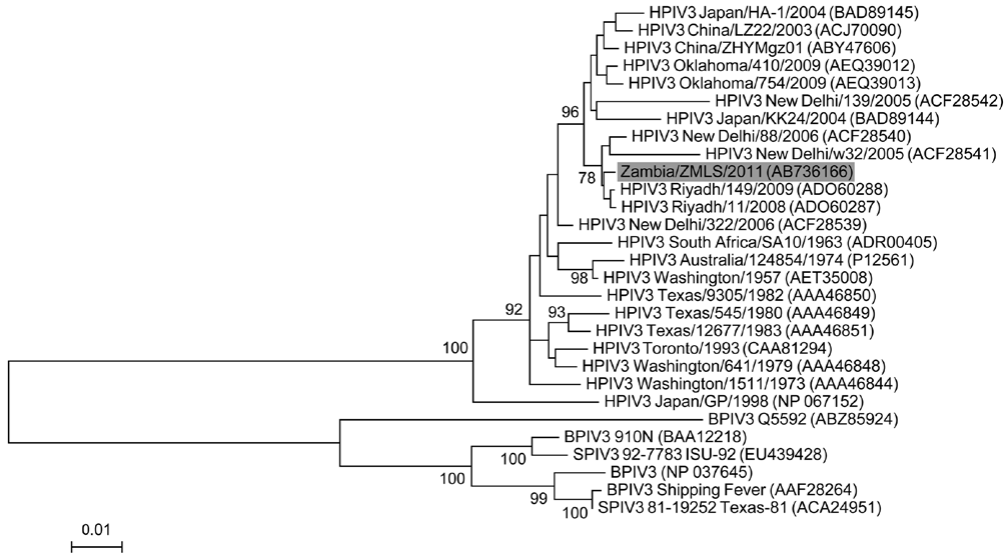
Phylogenetic analysis of the amino acid sequence of the HN protein of human parainfluenza virus type 3 (HPIV3). The phylogenetic tree was constructed on the basis of the deduced amino acid sequence of the full-length HN gene of ZMLS/2011 (gray shading) and known paramyxoviruses. GenBank accession numbers are given in parentheses. Significant bootstrap values (>70%) are shown. Scale bars indicate amino acid substitutions per site. SPIV, simian parainfluenza virus; BPIV, bovine parainfluenza virus.

To confirm and classify ZMLS/2011 as a strain of HPIV3, we amplified and sequenced the genome of ZMLS/2011 using total RNA sample positive for the RT-PCR screening. The obtained ZMLS/2011 sequence (15,298 bp) was deposited in the DDBJ database (GenBank/EMBL/DDBJ entry AB736166). The HPIV3 genome encodes 6 structural proteins, N, P, M, F, HN, and L. All the corresponding open reading frames were found in the ZMLS/2011 genome. A phylogenetic analysis was performed by using MEGA5 and based on the deduced amino acid sequence of the full-length HN protein (*10*). The phylogenetic tree clearly established ZMLS/2011 within the lineage of HPIV3 and distinct from other known parainfluenza viruses related to it (Figure 1). ZMLS/2011 is most closely related to HPIV3 strain Riyadh 149/2009, isolated from a hospitalized child in Saudi Arabia in 2009 (*11*). These results indicated that ZMLS/2011 identified from the chacma baboon is a strain of HPIV3.

**Figure 2:**
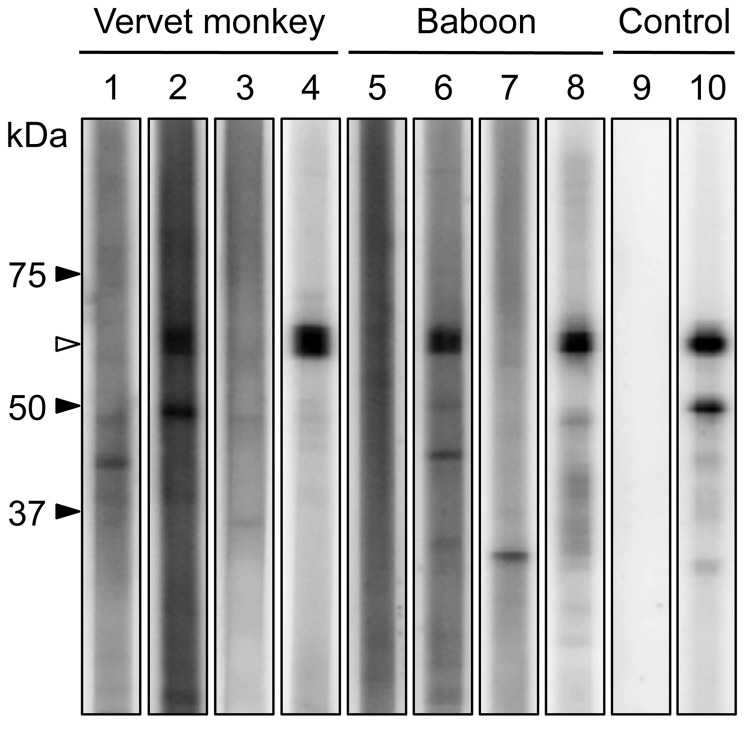
Western blot analysis of purified recombinant N protein from human parainfluenza virus type 3. Western blot analysis was performed by using serum specimens from vervet monkeys (lanes 1–4) and baboons (lanes 5–8) in the Mfuwe (lanes 1, 2, 5, 6) and Livingstone (lanes 3, 4, 7, 8) regions. Results of representative antibody-negative (lanes 1, 3, 5, 7) and antibody-positive (lanes 2, 4, 6, 8) samples are shown. Mock antibody (lane 9) and HPIV monoclonal antibody (lane 10) were used as negative and positive controls, respectively. The putative molecular mass of the recombinant N protein (white arrowhead) is 59 kDa.

Antibodies were detected by a recombinant N protein–based Western blot. Recombinant N protein of ZMLS/2011 was expressed in *Escherichia coli* and purified by histidine tag–based affinity chromatography. The 189 serum specimens were screened by using the Mini-PROTEAN II Multiscreen Apparatus (Bio-Rad, Hercules, CA, USA). Mouse monoclonal HPIV antibody (MAB819; Millipore, Billerica, MA, USA) served as the positive control. Among the serum samples tested, 2 (4%) from 50 yellow baboons, 11 (22%) from 50 chacma baboons, and 6 (7%) from 89 vervet monkeys had HPIV3 antibodies (Table; Figure 2). Positive results were obtained from serum samples collected from animals in the Mfuwe and Livingstone regions. All 4 baboons positive for the HPIV3 genome were negative for HPIV3 antibodies (data not shown), suggesting that, at the time the samples were taken, these HPIV3 antibody–negative baboons might have been in the acute stage of infection, before a detectable immune response had developed.

**Table:** Sample information and results of the molecular and serologic analyses of human parainfluenza virus type 3, Zambia

Animal (species)	Sampling location	RT-PCR positive/total*	Western blot positive/total
Yellow baboons (*Papio cynocephalus*)	Mfuwe	1/50	2/50
Vervet monkeys (*Chlorocebus pygerythrus*)	Mfuwe	0/50	3/50
Chacma baboons (*P. ursinus*)	Livingstone	3/50	11/50
Vervet monkeys (*C. pygerythrus*)	Livingstone	0/39	3/39
Total		4/189	19/189

*RT-PCR, reverse transcription PCR.

## Conclusions

We identified wild baboons positive for HPIV3 by using molecular and serologic analyses. Seropositive vervet monkeys also were found. These nonhuman primates are widely distributed in Africa (*8*), and their habitats overlap those of humans, mainly in rural areas. A survey performed in Kenya of humans with influenza-like illness and severe acute respiratory illness showed that 9.4% were positive for HPIVs (*12*). However, HPIV3 infection in humans in Africa has been poorly studied, making ZMLS/2011 difficult to compare with an epidemic strain of HPIV3 among humans. The nonhuman primates sampled in this study live side by side with humans. In addition, many tourists visit the Livingstone region and, in some instances, are harmed by the nonhuman primates attempting to grab food carried by humans. Detection of HPIV3 in wild nonhuman primates might be related to these contacts. Further epidemiologic studies of humans and wild nonhuman primates are needed to determine whether HPIV3 is transmitted between humans and wild nonhuman primates.

Serologic evidence of HPIV3 infection was obtained from baboons and vervet monkeys in 2 distinct geographic areas of Zambia, but little is known about HPIV3 infection in wild nonhuman primates. In 1963, simian agent 10 (also known as simian virus 10) was isolated from the mouth of a Samango monkey (*Cercopithecus mitis*) in a laboratory in South Africa (*13*). Complete genome sequence analysis showed simian agent 10 as a strain of HPIV3 (*14*). Experimental infections showed that many nonhuman primates—including chimpanzees; macaques; and squirrel, owl, patas, and rhesus monkeys—are sensitive to HPIV3 infection (*1*,*15*). These previous reports support our finding that wild nonhuman primates are susceptible to HPIV3 infection.
